# Polysaccharide-based biomaterials for regenerative therapy in intervertebral disc degeneration

**DOI:** 10.1016/j.mtbio.2024.101395

**Published:** 2024-12-10

**Authors:** Xin Wang, Yixue Huang, Yilin Yang, Xin Tian, Yesheng Jin, Weimin Jiang, Hanliang He, Yong Xu, Yijie Liu

**Affiliations:** a Department of Orthopaedic Surgery, The Fourth Affiliated Hospital of Soochow University, Suzhou Medical College, Soochow University, Suzhou, 215000, China; b Department of Orthopedic Surgery, The First Affiliated Hospital, Orthopedic Institute, MOE Key Laboratory of Geriatric Diseases and Immunology, Suzhou Medical College, Soochow University, Suzhou, 215000, Jiangsu, China; c Department of Internal Medicine and Clinical Nutrition, Institute of Medicine, Centre for Bone and Arthritis Research at the Sahlgrenska Academy, University of Gothenburg, Gothenburg, 41346, Sweden

**Keywords:** Intervertebral disc degeneration, Polysaccharides, Biomaterials

## Abstract

Intervertebral disc (IVD) degeneration represents a significant cause of chronic back pain and disability, with a substantial impact on the quality of life. Conventional therapeutic modalities frequently address the symptoms rather than the underlying etiology, underscoring the necessity for regenerative therapies that restore disc function. Polysaccharide-based materials, such as hyaluronic acid, alginate, chitosan, and chondroitin sulfate, have emerged as promising candidates for intervertebral disc degeneration (IVDD) therapy due to their biocompatibility, biodegradability, and ability to mimic the native extracellular matrix (ECM) of the nucleus pulposus (NP). These materials have demonstrated the capacity to support cell viability, facilitate matrix production, and alleviate inflammation in vitro and in vivo, thus supporting tissue regeneration and restoring disc function in comparison to conventional treatment. Furthermore, polysaccharide-based hydrogels have demonstrated the potential to deliver bioactive molecules, including growth factors, cytokines and anti-inflammatory drugs, directly to the degenerated disc environment, thereby enhancing therapeutic outcomes. Therefore, polysaccharide-based materials provide structural support and facilitate the regeneration of native tissue, representing a versatile and effective approach for the treatment of IVDD. Despite their promise, challenges such as limited long-term stability, potential immunogenicity, and the difficulty in scaling up production for clinical use remain. This review delineates the potential of various polysaccharides during the fabrication of hydrogels and scaffolds for disc regeneration, guiding and inspiring future research to focus on optimizing these materials for clinical translation for IVDD repair and regeneration.

## Graphical abstract

Summary of polysaccharide-based biomaterials for intervertebral disc regeneration and repair.

**Figure:**
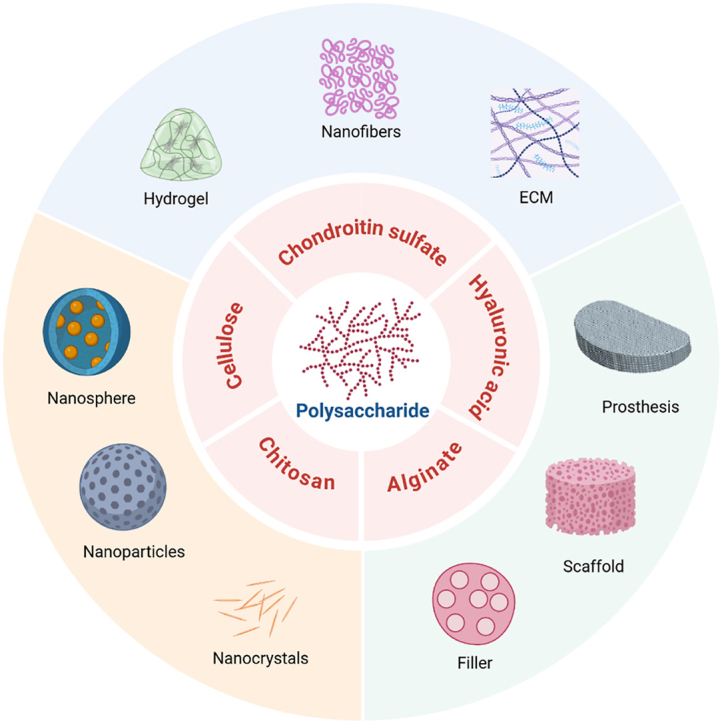

## 1 Introduction

Low back pain (LBP) presents a remarkably prevalent affliction and has been the leading cause of years lived with disability (YLDs) [1]. In 2020, an estimated 619 million people worldwide were affected by low back pain, and the number is estimated to be 843 million (759–933) by 2050 [2]. Various elements including vertebrae, sacroiliac joint, intervertebral disc, neurovascular structure, and soft tissue can alone or jointly result in low back pain [3]. Intervertebral disc degeneration (IVDD), which contributes to spinal canal stenosis, spondylolisthesis, and deformity, has been identified as one of the major causes of LBP [4]. Notably, annual economic costs related to IVDD exceed US$100 billion in the United States [5] (see Table 1, Table 2, Table 3, Table 4, Table 5).

**Table 1:** Applications of chondroitin sulfate-based biomaterial in IVDD regeneration and repair.

Material	Biological factor, agent or cell	Preparation method	Type of study	Function and advantage	Ref
Hyaluronic acid (HA), tetra poly (ethylene glycol) with maleimide end groups (Teran-PEG-MAL), and chondroitin sulfate (CS)		Ex situ solution penetration	In vitro, in vivo (rat)	Injectable anti-inflammatory hydrogel, with Diels-Alder reaction-mediated sequential crosslinking enhanced mechanical property and pH-responsive release function	[15]
Chondroitin sulfate, poly (ethylene glycol)-*b*-poly (trimethylene carbonate)-acrylate (PEG-*b*-PTMC-Ac)	Bovine NPCs	Ex situ solution penetration	In vitro	In situ forming, mechanically resilient hydrogel, with high NPCs viability and ECM production.	[73]
poly (N-isopropylacrylamide)-graft-chondroitin sulfate (PNIPAAm-g-CS), alginate microparticles	Growth differentiation factor 6 (GDF-6)	Solution mix	Ex vivo, in vitro	In situ forming hydrogel, enhanced initial injectability, bioadhesiveness, and mechanical performance.	[74,75]
Decellularised extracellular matrix of bovine coccygeal discs functionalized with chondroitin sulfate		Solution mix	In vitro	Injectable, self-assembled biomimetic hydrogel, boosting sGAG production and preserving rounded cell morphology of nasal chondrocytes	[72]

**Table 2:** Applications of hyaluronic acid-based biomaterial in IVDD regeneration and repair.

Material	Biological factor, agent or cell	Preparation method	Type of study	Function and advantage	Ref
Hyaluronic acid-1,4-butanediol diglycidyl ether (HA-BDDE), HA-poly (N-isopropylacrylamide) (HA-pNIPAM)	NP cells	In situ penetrating	In vitro, ex vivo (bovine IVD)	Support high cell viability, facilitate ECM production, and withstand confined cyclic axial compression.	[84]
Type Ⅰ collagen, tyramine-substituted hyaluronic acid	BM-MSCs	Solution mix	In vitro	Promote NP ECM biopolymers, increase hydration degree, and ensure cell adhesion and survival.	[85]
Fibrin, collagen, and hyaluronic acid		Solution mix	In vitro	Maintain cell viability, and increase the sGAG to collagen ratio.	[86]
Hyaluronic acid, zirconium oxide		Nanoparticles addition into solution	In vivo (goat) and ex vivo (rabbit)	Real-time visualization and monitoring, support disc height and promote anabolism of IVD	[87]
Polyethylene glycol, hyaluronic acid, PPS	Mesenchymal precursor cells	Ex situ penetrating	In vitro	Injectable, enzymatically-crosslinked hydrogel, enhance MPCs differentiation to NP-like tissue.	[89,90]
HA and poly (N-isopropylacrylamide) (pNIPAM)	Stromal cell-derived factor 1 (SDF-1)	Direct amidation reaction	Ex vivo (bovine IVD)	Increase both the number of recruited cells and their migration distance within the intervertebral disc (IVD) tissue.	[91]
Collagen, HA, and gelatin microspheres	Transforming growth factorβ3 (TGF-β3)	Ex situ penetration	In vitro and in vivo (mouse)	possess superior viscoelastic properties and injectability, support MSC and NC differentiation	[92]
HA, butanediol diglycidyl ether		In situ crosslinking	In vitro and in vivo (rabbit)	Injectable, restore the disc hydration and function	[93]
Aldehyde hyaluronic acid, poly (amidoamine)	siRNA	Solution mix	In vitro and in vivo (rat)	Transport siSTING and silence STING signaling pathway in NPC, alleviate IVD inflammation and degeneration	[95]
Methacrylated hyaluronic acid microspheres	circSTC2 silencing gene vectors	Microfluidics	In vitro and in vivo (rat)	Silence circSTC2 expression in NPCs to maintain the ECM metabolism balance in the nutrient-restricted microenvironment and hinder IVDD	[96]
Oxidized and methacrylated glycosaminoglycan, fibrin, and poly (ethylene glycol) diacrylate (PEGDA)		In situ solution penetration	In vitro and ex vivo (bovine)	Injectable two-part hydrogel with high adhesion strength, low cytotoxicity, and mechanical compliance	[98]
Thiolated HA and PEG vinylsulfone		Ex situ penetration	In vitro	HA with lower molecular weight is advantageous for maximizing cell viability and sGAG synthesis.	[99]
Hyaluronic acid, collagen		In situ crosslinking	Ex vivo (rat)	Restore disc hydration and demonstrate superior instantaneous and equilibrium moduli	[100]

**Table 3:** Applications of cellulose-based biomaterial in IVDD regeneration and repair.

Material	Biological factor, agent or cell	Preparation method	Type of study	Function and advantage	Ref
Carboxymethylcellulose		Esterification of hydroxyl groups	In vitro	Injectable, photocrosslinked, and water-soluble hydrogel, with NP cells viable and mechanical integrity	[106]
Carboxymethylcellulose (CMC) and methylcellulose (MC)		Esterification of hydroxyl groups	In vitro, ex vivo (bovine caudal IVDs), in vivo (rat)	Thermoresponsive, tunable, biocompatible hydrogel with long-term structurally stable	[107,108]
Carboxymethylcellulose	Human mesenchymal stem cells (hMSCs)	Esterification of hydroxyl groups	In vitro	Photocrosslinked and biocompatible hydrogel, NP ECM elaboration, improved mechanical properties	[[109], [110], [111]]
Chitosan carboxymethyl cellulose	Adipose-derived stem cells (ASCs)	Ex situ solution penetration	In vivo (ovine), in vitro	Long-term stabilization of injectable composite hydrogel in degenerated IVDs in a large animal model	[112]
Poly (ethylene glycol) dimethacrylate and nano-fibrillated cellulose		Ex situ solution penetration	Ex vivo (bovine IVDs)	In situ injection and photopolymerization, restore IVD height and maintain after 0.5 million loading cycles	[113]
Methacrylated gellan-gum reinforced with cellulose nanocrystals		Solution mix	In vitro	Injectable composite hydrogel with improved scaffold stiffness that augments matrix entanglement	[114]
Cellulose-alginate double network hydrogel-based annulus fibrosus and cellulose hydrogel-based nucleus pulposus	Growth differentiation factor-5, SKP peptide (MSC homing peptide) and RGD peptide (cell adhesion peptide)	In situ addition	In vitro and in vivo (rats)	Recruit and induce MSCs differentiation to NP-like phenotype, has high compressibility, shape memory property and comparable mechanical strength	[115]
Cellulose nanofiber-based AF and type Ⅱ collagen-based NP	Rat NPCs and AFCs	Bioprinted bacterial cellulose by the fermentation of Acetobacter xylinum on a micropatterned template	In vitro and in vivo (rats)	Mimic native IVD and manifest distinguished structural and functional performance	[116]

**Table 4:** Applications of alginate-based biomaterial in IVDD regeneration and repair.

Material	Biological factor, agent or cell	Preparation method	Type of study	Function and advantage	Ref
Alginate, collagen-cryogel, hyaluronic acid		Froze and lyophilize alginate and collagen, crosslinked with EDC/NHS	In vivo (rat)	Ameliorate mechanical allodynia, preserve IVD hydration, and maintain the structure integrity of the disc.	[129]
Alginate		In situ crosslink by calcium carbonate and glucono-δ-lactone	In vitro, ex vivo (bovine caudal IVDs)	Reestablish function by reducing height loss during long-term cyclic loading and remains contained within the disc with no leakage at the injection site	[121]
Ultra-purified alginate	BMSCs, bone marrow aspirate concentrate (BMAC)	In situ gelling	In vitro, in vivo (rabbits)	Hinder IVD degeneration in histological grade and promote the repair of IVD defects.	[122]
Alginate beads	Human NPCs, Wharton's jelly MSC-derived extracellular vesicles	Solution mix	In vitro	Preserve cell viability, promote ECM deposition while inhibiting inflammation.	[123]
Alginate, poly (ethylene glycol) diacrylate (PEGDA) microcryogels	Mesenchymal stem cells (MSCs)	Ex vivo solution penetration	In vitro, in vivo (dog), ex vivo (canine IVD)	Injectable hydrogel with enhanced elasticity, alleviate IVDD in an ex vivo organ culture model and an in vivo canine model.	[124]
Alginate	Syndecan-binding peptides (AG73), integrin-binding peptides (cyclic RGD)	Strain Promoted Azide Alkyne Cycloaddition (SPAAC) click chemistry, maleimide-thiol click chemistry, solution mix.	In vitro	cRGD and AG73 loaded alginate hydrogel, contribute to higher cell viability, adhesion, biosynthetic property, and NPC phenotype expression.	[26]
Alginate, poly (N-isopropylacrylamide) (PNIPAAm), silicate ceramics	NPCs	Solution mix, in situ crosslinking	In vitro, in vivo (rat)	Thermosensitive, immunoregulatory, promote matrix synthesis.	[127]
Mesoporous bioactive glasses, sodium alginate	melatonin	Solution mix	In vitro, in vivo (rat)	Possess favorable physical and mechanical properties, and alleviate IL-1β triggered oxidative stress and inflammation.	[128]
Ultra-purified alginate	Human IVD cells	Solution dilution	In vitro, in vivo (sheep and rabbit)	Reduced cytotoxicity, favorable biomechanical property, and anabolism enhanced phenotype.	[131]
Ultra-purified alginate	Rabbit BMSCs	Solution dilution	In vitro, in vivo (rabbit)	Upregulate ECM and phenotypic marker, augment IVD regeneration.	[132]
Ultra-purified alginate	Rapidly expanding clones of human MSCs	Solution mixture	In vivo (rat)	Suppress inflammatory cytokine production, reduce nociceptive behavior of rats.	[133]
Sodium alginate, gelatin	Antagomir-204–3p	Solution mix	In vitro, in vivo (	Improve IVD mechanical property, sustain hydration, and preserve structural integrity.	[130]
Oxidized alginate microbeads, fibrin	RGD peptides, AFCs	Genipin-crosslinking, ex situ solution penetration	In vitro, ex vivo (bovine IVD)	Preserve high cell viability, inhibit AFCs apoptosis, reduce herniation risk in bovine caudal IVD organ for a long-term.	[135]
Alginate	IL-1 receptor antagonist (IL-1Ra), soluble tumor necrosis factor receptor-1 (sTNFR1)	Cell encapsulation in solution	In vitro, ex vivo (human cadaveric IVD)	Inhibit IL-1β and TNF expression, promote proteoglycan and collagen production	[136]
Alginate, poly (ε-caprolactone) microfibers	AFCs and NPCs	Ex situ fabrication	In vitro, in vivo (rat)	Enhance ECM deposition and mechanical properties.	[137]

**Table 5:** Applications of chitosan-based biomaterial in IVDD regeneration and repair.

Material	Biological factor, agent or cell	Preparation method	Type of study	Function and advantage	Ref
Chitosan-glycerophophate	NP cells	Solution mix	In vitro	Thermosensitive and biocompatible hydrogel that retains proteoglycan of NP cells	[142]
Chitosan-glycerophosphate	Mesenchyme stem cells	Solution mix	In vitro	Temperature-sensitive hydrogel, capable of inducing proteoglycans and collagens production of MSCs	[143]
Chitosan-glycerophosphate (SHC0.075BGP0.1)	NP cells	Solution mix	In vitro, ex vivo (human cadaveric IVDs)	Similar mechanical properties to the human NP tissue, induction of highest GAG	[144]
*N*-hexanoyl glycol chitosan		*N*-hexanoylation reaction of glycol chitosan	In vitro, in vivo (rat), ex vivo (pig)	Thermos-sensitive injectable hydrogel with no cytotoxicity and no adverse effects in a rat model, maintaining the stability of defective porcine IVD for 28 days.	[145]
Chitosan and poly-(γ-glutamic acid nanoparticles	diclofenac	Ex situ solution penetration	In vitro, in vivo (rat)	Ch/Df/γ-PGA NPs inhibits pro-inflammatory mediator production and reverse ECM synthesis.	[[147], [148], [149]]
Methacrylate chitosan and aldehyde polyethylene glycol		Solution mix, Schiff base reaction	In vitro, in vivo (rat)	Rapidly in situ forming injectable hydrogel with low cytotoxicity and enhanced mechanical strength.	[150]
Chitosan, gelatine	Link N	Solution mix	In vitro	Improved mechanical properties in compression and pro-anabolism function	[151]
Chitosan, poly (l-lactic acid)	Human umbilical cord mesenchymal stem cells (hUCMSCs)	Solution mix	In vivo (rabbit)	Beneficial for cell adhesion and proliferation, enhance bone connection between adjacent vertebrae and promote AF tissue regeneration	[152]
Chitosan with dissolved perfluorotributylamine (core), polycaprolactone (shell)	Annulus fibrosus stem cells (AFSCs)	Coaxial electrospinning, solution mix	In vitro, in vivo	Persistent oxygen release, promoting proliferation, migration and ECM production of AFSCs.	[154]
Chitosan, poly (butylene succinate-co-terephthalate) (PBST), poly (ether ether ketone) (PEEK)	IVD cells	In vitro fabrication	In vitro, in vivo (pig)	Support IVD cell growth, possess a good compressive stress and elastic moduli.	[155]
Chitosan, poly (butylene succinate-co-terephthalate) copolyester	AF and NP cells	In vitro fabrication	In vitro, in vivo (rabbit)	Maintain the biological function of IVD cells and retain the height of intervertebral	[156]

IVDD is a complex and multifactorial process that involves biochemical, structural, and mechanical changes in the disc tissue. The pathological process includes (a) progressive cell senescence and cell death [6], (b) cell dysfunction and declined anabolic production of extracellular matrix (ECM) [7], (c) inflammatory cells and cytokines, and increased catabolic activity [8]. The loss of hydration and structural integrity accelerates the biomechanical property to fail and results in pain and disability. Furthermore, there exist complex interactions between the degenerated disc, adjacent vertebral endplates, and the paraspinal muscles. Changes in the endplates can alter nutrient transport to the disc, exacerbating degeneration, while the paraspinal musculature may undergo compensatory changes, further influencing spinal biomechanics and pain perception [9]. Understanding these interconnected factors is critical for developing holistic therapeutic strategies.

Although progress in understanding the molecular basis of IVDD has been made, the current treatments for IVDD, either conservative or operative options, are limited. Non-surgical treatments including immobilization, analgesic drugs, and other physical therapy, mainly aim to alleviate the pain [10]. In severe cases, invasive surgical treatments like interbody fusion or arthroplasty are necessary to provide mechanical structural support and stability [11,12]. Although pain control can be beneficial for patients, the long-term prognosis of anti-inflammatory drug use is not optimal. Additionally, the risk of adjacent segment degeneration (ASD) exists with invasive surgical procedures [13]. Essentially, conservative treatments or surgical options can not address the underlying etiology or facilitate the regeneration of the degenerated disc tissue. In comparison, polysaccharide-based biomaterials possess the potential to alleviate the inflammatory microenvironment [14], halt cell senescence and apoptosis [15], improve biomechanical instability [16], and prevent IVD from degeneration. This is beneficial for addressing IVDD from the etiological source.

Since traditional treatments could not reverse degenerative progress, restore tissue homeostasis, and reconstruct the mechanical stability of the spine. New strategies aimed to counteract intervertebral disc degeneration are urgent. Many attempts have been made in the following fields. (a) anti-inflammatory strategies to regulate the inflammatory microenvironment [17,18]; (b) vascularization and innervation growth inhibition [19]; (c) anti-catabolic strategies [20]; (d) pro-anabolic strategies [21]; (e) endogenous and exogenous cell supplementation [22,23]. The main purposes of ingenious therapeutics are to alleviate inflammation in IVD which contributes to discogenic pain and degenerative symptoms and to remodel anabolic/catabolic balance in the nucleus pulposus (NP). Among these regenerative approaches, emerging materials such as synthetic hydrogels, decellularized tissue matrices, peptide-based scaffolds, and stimuli-responsive smart materials have shown promise in supporting disc cell viability, mimicking the disc's extracellular matrix, and enhancing mechanical resilience [[24], [25], [26], [27]]. Advanced cellular approaches, including stem cell therapies, gene editing, and endogenous or exogenous biological factor recruitment are also under investigation to directly repair or regenerate damaged tissue [8,[28], [29], [30]]. Against this backdrop, polysaccharide-based biomaterials stand out due to their biocompatibility, tunable mechanical properties, and ability to interact with native disc cells and matrix components.

In situ IVD injection has become the paradigm for drug delivery in that systematical administration can barely reach the disc due to avascularity and immune privilege. However, puncture itself is an invasive procedure that may impair annulus fibrosus (AF) and therefore facilitate IVDD [31] and the simply-delivered drugs are susceptible to transient clearance, limited absorption by nucleus pulposus cells (NPCs), and off-target side effects [32]. In such a situation, biomaterial delivery systems based on tissue engineering hold promise in IVD repair and regeneration. The bioinspired delivery platform can not only bear the mechanical pressure of the IVD but also improve drug efficacy due to their excellent biocompatibility, biodegradability, accurate tissue targeting, and sustained release [33], of which polysaccharide-based materials are ideal candidates [34].

## 2 Intervertebral disc degeneration

### 2.1 Anatomy and physiology of intervertebral disc

**Fig. 1:**
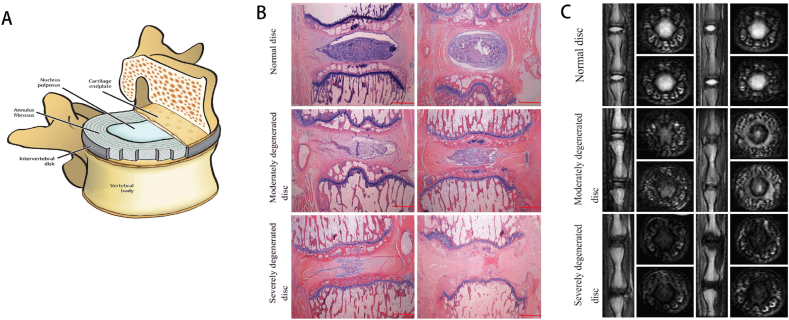
(A) A schematic view of the human IVD (Adapted from Ref. [38]). (B and C) Representative H&E staining and representative T2-weighted MRI scans of disc samples from normal, moderately degenerated and severely degenerated groups.

The intervertebral disc, a fibrocartilaginous joint between adjacent vertebrae, consists of three main components: the gelatinous NP as the core, the annulus fibrosus surrounding the periphery, and the cartilage endplates (CEP) sandwiching aforesaid two structures [35]. The NP contains two types of cells, NPCs and notochordal cells, the latter of which only exist in the embryonic and fetal stages. Its extracellular matrix (ECM) is defined by a network of irregularly organized type Ⅱ collagen embedded in amorphous proteoglycans [25]. The AF is made up of 15–25 concentric layers of type I collagen fibers, with inner and outer regions, primarily responsible for resisting tensile forces [36] (Fig. 1). The upper and lower CEPs, through which the main source of nutrition is diffused and metabolites are excreted, interface the vertebral body and the disc [37].

Proteoglycans are proteins with glycosaminoglycan chains covalently attached. The glycosaminoglycan chains endow aggrecan osmotic properties to swell and withstand compressive pressure, to which the remarkable chondroitin sulfate (CS) and keratin sulfate (KS) chains are ascribed [39,40]. Of note, as a key constituent of ECM (65 % in NP and 20 % in AF), aggrecan is essential in endowing IVD mechanical property to resist axial compression in that negatively charged glycosaminoglycans strongly attract water molecules and bind growth factors and cell adhesion molecules [40]. Recent advances have demonstrated that aggrecan is critical for the preservation of appropriate stiffening of the ECM by providing biomechanical cues during IVD development [41]. In addition, aggrecan restrains endothelial cell adhesion and cell migration, therefore helping maintain the avascular condition as well as inhibiting nerve growth [42]. In particular, chondroitin sulfate (CS), as one kind of glycosaminoglycan, contributes to hydration, cell survival, and the non-innervated state of IVD [43]. With growth and development, glycosaminoglycan chains undergo several changes including that the CS shortens while the KS elongates, accompanied by sulfation site transferring from the 4-carbon to the 6-carbon of N-acetyl galactosamine [44].

### 2.2 Pathophysiology and comprised biomechanism of IVDD

**Fig. 2:**
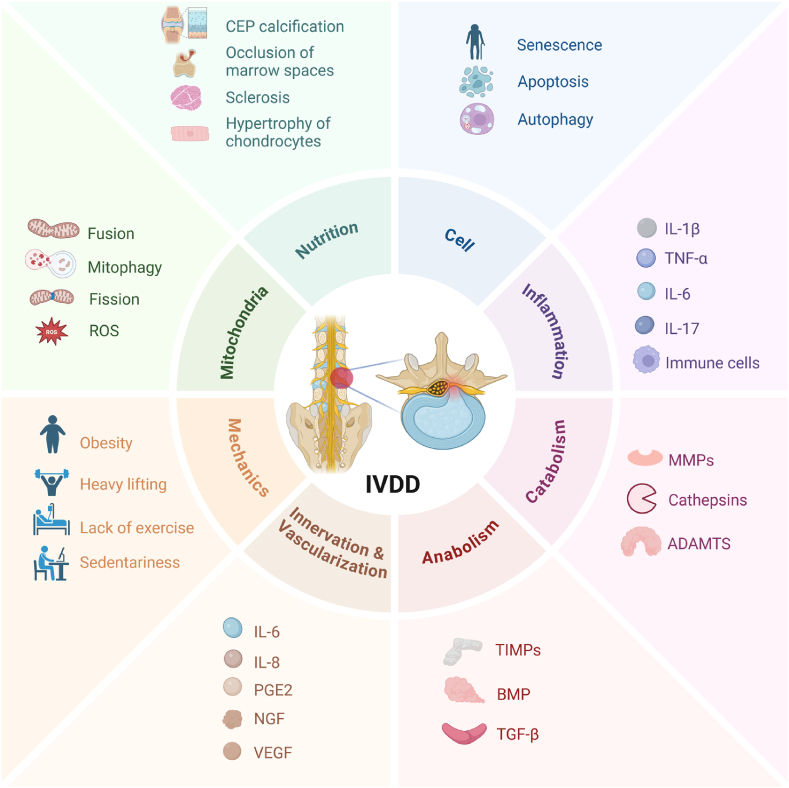
Schematic representation of biomechanical causes, biological factors and microenvironment changes in the process of IVDD. Multifactorial changes are involved in IVDD, including cellular changes, anabolism/catabolism imbalance, inflammatory response, mitochondrial dysfunction, calcification and endplate changes, and altered biomechanics. Loss of notochordal cell marks the beginning of the degeneration process, followed by nucleus pulposus cell decrease. Reduced matrix production and aggravated matrix degradation define phenotypic changes, leading to disc dehydration and loss of height. Pro-inflammatory cytokines upregulation and immune cell infiltration exacerbate disc microenvironment. Neovascularization and nerve infiltration are associated with discogenic pain. Mitochondrial dysfunction including accumulated ROS, impaired mtRNA, and altered mitochondrial dynamics contributes to and perpetuates disc degeneration. Endplate calcification and subchondral sclerosis limit nutrient supply to disc. IL-1β: Interleukin 1β, IL-6 and IL-17: Interleukin 6 and 17, TNF-α: Tumor necrosis factor α, PGE2: Prostaglandin E2, MMPs: Metalloproteinases, TIMPs: Tissue inhibitors of metalloproteinases, BMP: Bone morphogenetic protein, TGF-β: Transforming growth factor β, NGF: Nerve growth factor, ADAMTS: A disintegrin and metalloproteinase with thrombospondin motifs, VEGF: Vascular endothelium growth factor, ROS: Reactive oxygen species, mtRNA: mitochondrial RNA.

IVDD defines the crucial pathological basis of degenerative disc disease (DDD), triggered by multiple factors including genetics, environmental factors, and mechanical factors [45] (Fig. 2). Cytokines, for example, IL-1β and TNF-α, aggravate the degenerative process and promote cellular senescence, autophagy, and apoptosis. The inflammatory cascade mediates catabolism through upregulating extracellular matrix enzymes such as MMP3 and ADAMTS production and restrains anabolism by inhibiting proteoglycan expression [[46], [47], [48]]. The reduction of proteoglycans is the most prominent biochemical variation and is recognized as a hallmark of IVDD [49]. With proteoglycan and type-Ⅱ collagen collapsing, the strength-bearing capacity of IVD and biomechanical integrity was impaired, leading to AF tear and subsequent NP exposure to the circulatory system and immune system [45]. In the meantime, evoked inflammatory cytokines, IL-6, prostaglandin E2 (PGE2), and pro-innervating factors nerve growth factor (NGF) stimulate nerve ingrowth, leading to symptomatic pain [47,50]. Upregulated vascular endothelium growth factor (VEGF) and chemokines, CCL1, 3 and 13, CXCL10 by NPCs provoke vascularization and immune cells infiltration, which accumulates the inflammatory cytokines including IL-1β and TNF-α, aggravating catabolism and ECM loss [47,51,52]. In addition, CEP calcification evolves with aging, breaking the supply/demand balance. The nutrient diffused inclines while demand rises due to increased infiltrated immune cells, which accelerates the disorder of energy metabolism [53]. This imbalance restrains the nutrients for NPCs and annulus fibrosus cells (AFCs) and further affects cell viability. Furthermore, a deteriorating extracellular environment characterized by significant acidification with metabolite cumulating induces further cell senescence and apoptosis and aggravates degradative enzyme production [54].

During IVDD, proteoglycan in NP undergoes a significant decrease and leads to the ratio of NP/AF proteoglycan dropping, which indicates the importance of high proteoglycan concentration in healthy IVD [49]. Hyaluronic acid, as another major component of proteoglycans with aggrecan monomers linked, imbibes water and subsequently renders IVD mechanical strength. In addition, hyaluronan demonstrates an anti-inflammatory property by inhibiting tetratricopeptide repeats 3 (IFIT3) and insulin-like growth factor binding protein-3 (IGFBP3) and a matrix remodeling action by improving anabolism of extracellular matrix and reducing ADAMTS4 [14]. Although the content of aggrecan declines in NP during aging, there is a compensating rise of aggrecan concentration in the inner AF [55]. Furthermore, as MMPs and aggrecanases cleave aggrecan, whether fragmented aggrecan is harmful to disc needs to be elucidated.

Mitochondrial dysfunction is associated with the development of IVDD through affecting a series of changes including mitochondrial fusion, autophagy, and cell apoptosis [56,57] (Fig. 2). Interestingly, hyaluronic acid can preserve mitochondrial function via activating mitophagy in NPCs, thus attenuating IVDD [58]. Conversely, free hydroxyl radicals from hydrogen peroxide and superoxide are detrimental for HA to depolymerization [59].

## 3 Overview of polysaccharides

Polysaccharides are made up of monosaccharides and connected by O-glycosidic bonds. Widespread in nature, a variety of sources of polysaccharides includes algae (agarose), plants (starch, pectin), microorganisms (xanthan gum, gellan gum) and animals (chondroitin, heparin, hyaluronan) [60]. With regard to their chemical structure, the various functional groups of different polysaccharides lay the foundation for physicochemical modifiability, so as to endow more characteristics and functionalities [61]. In addition to the similarities between polysaccharides and the extracellular matrix (ECM), polysaccharides exert essential biological roles in molecular recognition, cell adhesion, and signal transduction [62]. Therefore, considering their impressive merits including biocompatibility, biodegradability, hydrophilicity, nontoxicity, bioactivity, and water retention capacity, polysaccharides have garnered significant attention for diverse biomedical applications including drug delivery, tissue repair, as well as targeted therapy [34]. Several common forms of polysaccharide-based biomaterials include hydrogels (e.g. hyaluronic acid, chondroitin sulfate, and alginate), scaffolds (e.g. chitosan and cellulose), nanoparticles (e.g. chitosan, hyaluronic acid, and alginate), microspheres (e.g. alginate and chitosan), nanofibers (chitosan, collagen, and silk fibroin), and nanocrystals (e.g. cellulose). These forms of polysaccharide-based materials provide a versatile toolkit for addressing different aspects of IVDD.

### 3.1 Chemical properties

As a polymeric carbohydrate, a polysaccharide is composed of more than 10 monosaccharides, among which the same monosaccharides constitute homopolysaccharides while different monosaccharides constitute heteropolysaccharides. According to the glycosidic linkage type and electrical charges, the polysaccharides can be classified into straight or branched chains and polyanion, electroneutral, or polycation polysaccharides respectively [63].

Polysaccharides that derive from natural sources have their own drawbacks including microorganism pollution, batch variation, different hydroxide content, and unstable mechanical strength [64]. However, the chemical modifications minimize these limitations and impart versatile functionalization to polysaccharides. There are diverse approaches including carboxymethylation, oxidation, sulfation, and polymer graft to achieve this transformation.

### 3.2 Crosslinking mechanisms of polysaccharides

Hydrated polysaccharides in an aqueous environment define the process of production of the hydrogel. As such, one of the most extensive usages of polysaccharides in tissue engineering and biomedical applications is hydrogel establishment. The mechanism whereby polysaccharides form network structure is mainly divided into physical crosslinking that is defined by ionic interaction, of which alginate represents the paradigm and chemical crosslinking that includes radical polymerization (e.g. dextran), aldehyde (e.g. amine contained polysaccharides), addition reaction, and condensation reaction.

## 4 Biomedical applications of various polysaccharide-based materials in IVDD

Mammalian polysaccharides including hyaluronic acid, keratan sulfate, and chondroitin sulfate naturally exist in vivo and constitute the ECM of the intervertebral disc. Glycosaminoglycan chains covalently attach to core protein and compose proteoglycans, contributing to structure integrity and microenvironmental homeostasis of intervertebral discs. In the following section, the fabrication and application of polysaccharide-based materials and biological properties in intervertebral disc repair and regeneration will be discussed.

### 4.1 Chondroitin sulfate

Widely distributed in vertebrates, invertebrates, and bacteria, chondroitin sulfate is characterized as a linear polysaccharide composed of repeated disaccharides of N-acetyl galactosamine and glucuronic acid. In regard to different sulfated residues at carbon positions, CS is classified into CS-A (chondroitin-4-sulfate), CS-C (chondroitin-6-sulfate), CS-D (chondroitin-2,6-sulfate) and CS-E (chondroitin-4,6-sulfate) [65]. There has been reports that CS significantly inhibited NF-κB activation and nuclear translocation, along with the production of inflammation cytokines and inducible nitric oxide synthase [66]. As a prominent constituent of the ECM, apart from anti-inflammatory, anti-oxidant, anti-coagulating, and immunomodulatory functions, CS serves as a signaling molecule that can influence cell signal transmission and promotes the formation of collagen and proteoglycans [67,68]. Moreover, CS has been reported to promote ECM production via the integrin-WNT5A pathway and notochordal cells-derived CS has been observed to inhibit hyperinnervation and peripheral nociception [69,70]. CS is now extensively utilized in biomedical applications because of its renowned compatibility with biological systems, biodegradability, and hydrophilicity [71].

#### 4.1.1 Applications of chondroitin sulfate-based materials in NP regeneration

**Fig. 3:**
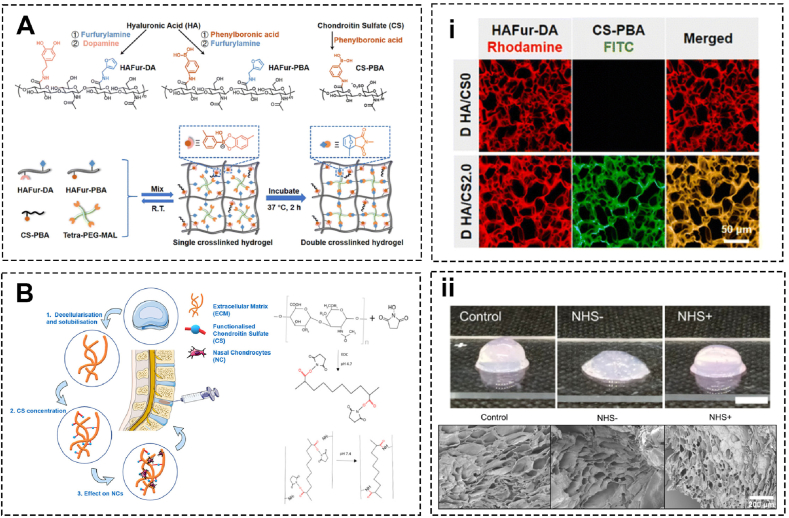
Chondroitin sulfate-based biomaterials for intervertebral disc regeneration and repair. (A) Injectable self-antioxidant hydrogels grafted with inflammation inhibitory chondroitin sulfate formed via dynamic boronate ester bonding between furan/phenylboronic acid and furan/dopamine-modified hyaluronic acid and mechanically enhanced by Diels-Alder reaction-induced secondary crosslinking, and (i) CLSM images of double-crosslinked HA/CS0 and HA/CS2.0 hydrogels (Adapted from Ref. [15]). (B) Injectable Disc-Derived ECM Hydrogel functionalized with chondroitin sulfate via amine groups of collagen and (ii) Assessment of the structural stability of cell free ECM-derived hydrogels and SEM images (Adapted from Ref. [72]).

Based on the anti-inflammatory properties of CS, Luo et al. engineered a versatile hydrogel system (HA/CS). This hydrogel is injectable and remarkably self-crosslinked post-injection through boronate ester bonds. Further enhancement of mechanical strength through sequential coupling through the Diels-Alder reaction to simulate the human NP. CS can degrade under physiological stimuli such as hyaluronidase enzyme, reactive oxygen species, and pH change, thus enabling site-specific release. As such, this hydrogel system continuously protects NPCs from apoptosis and preserves extracellular matrix synthesis. In the rat model, the HA/CS hydrogel inhibits inflammatory cascade and remarkably alleviates IVDD [15] (Fig. 3A).

A prospective treatment attempt involves resecting the compromised NP tissue and restoring it with NPCs encapsulated in situ forming hydrogel. Riahinezhad et al. designed such a hydrogel using thiolated chondroitin sulfate crosslinked 4-arm [poly (ethylene glycol)-b-poly (trimethylene carbonate)-acrylate] via Michael type reaction. This hydrogel delivers NPCs with high viability and proliferating ability. These cells exhibit collagen type Ⅱ deposition instead of collagen type Ⅰ. The instantaneous moduli of this hydrogel are adjusted according to the native NP and resistant to cyclic compressive loading [73].

An innovative graft copolymer, poly (N-isopropyl acrylamide)-graft-chondroitin sulfate (PNIPAAm-g-CS) combines the thermoresponsive behavior of the latter with the biocompatibility and bioactivity of CS to form a moisture-absorbing and fluid substance below the lower critical solution temperature and precipitated hydrogel above it [74]. However, this combination has inadequate solution viscosity and adhesiveness. Christiani et al. embedded alginate microparticles into PNIPAAm-g-CS to enhance surface roughness and toughness. The structure provides an optimized platform for sustaining, multiplying, and differentiating adipose-derived stem cells (ADSCs) into native NP-mimicking phenotype due to incorporated GDF-6, a potent chondrogenic factor. The ex vivo implantation of this formation into degenerated porvine IVDs demonstrated promising results in restoring the compressive and neutral zone stiffness to levels seen in undamaged discs [75].

Chondroitin sulfate and type Ⅱ collagen constitute the major ECM of NP. Borrelli et al. designed a self-assembled hydrogel of decellularized ECM functionalized with chondroitin sulfate. Of note, the inclusion facilitates a rounded morphology and higher sGAG synthesis of nasal chondrocytes which have been recognized as a promising cell source for NP regeneration with their robust matrix synthesis capacity [72] (Fig. 3B). Basically, the effect of chondroitin sulfate on the chondrogenic phenotype of mesenchymal stem cells has been reported to be associated with Smad1/5/8 signaling inhibition and p38 signaling upregulation [76,77].

### 4.2 Hyaluronic acid

Hyaluronic acid (HA) is a nonsulfated, linear polyanion mucopolysaccharide and the only glycosaminoglycan that is ubiquitous in almost all mammalian animals, with additional production capabilities in certain bacteria [78]. This material is widely present in the extracellular matrix. It plays a vital part in diverse biological processes, including cell development, migration, as well as influencing the physiological activities of the human body [79]. Specifically, CD44, the best-characterized HA receptor, mediates cell migration of MSCs through interaction with extracellular HA, and its expression on activated lymphocytes aids in the primary rolling to the inflammatory sites in a HA-dependent manner [80]. Hyaluronic acid is a versatile and extensively used material in tissue engineering because of its biocompatibility, biodegradability, nonimmunogenicity, and viscoelastic properties [81]. Its applications span from scaffold fabrication and drug delivery platforms to wound care, ophthalmology, and orthopedics. The extensive study and successful utilization of HA and its derivatives underscore their considerable potential in advancing regenerative therapies and improving patient outcomes in various medical fields.

#### 4.2.1 Applications of hyaluronic acid-based materials in NP regeneration

One ideal biomaterial as a novel treatment for IVD regeneration is able to support the expansion, differentiation, and function of encapsulated cells while providing sufficient mechanical stability. It has been shown that HA grafted with poly (N-isopropyl acrylamide) supports high cell viability and maintains differentiation potential [82]. HA crosslinked with 1,4-butanediol diglycidyl ether (BDDE) has superior comprehensive mechanical testing performance [83]. Therefore, Guo et al. combine HA-BDDE and HA-pNIPAM to form an interpenetrating polymer network as a cell carrier. The IPN hydrogel demonstrates rheological properties similar to native NP tissue. This network promotes GAG production and new matrix deposition of loaded NPCs after implanted ex vivo in organ cultured IVD [84]. Using hyaluronic acid and type Ⅰ collagen to mimic the native nucleus pulposus tissue, encapsulated human BMSCs exhibit high gene expression of NP marker under a differentiation medium containing GDF5 and TGFβ1. Of note, cells tend to have a rounded morphology to NPCs [85]. Similarly, Gansau et al. incorporated collagen and hyaluronic acid into fibrin-based hydrogel. Cell viability and proliferation are enhanced due to the addition of ECM components. Furthermore, incorporating HA elevates sGAG accumulation and inhibits collagen expression [86]. Granular hydrogels have become favorable biomaterials with injectability and microscale porosity in tissue engineering. Muir et al. designed a hyaluronic acid granular hydrogel while encapsulating radiopaque zirconium oxide nano-powder. This radiopaque hydrogel can be monitored over time to track its location, degradation, and interaction with surrounding tissues, providing visualization of the placement of the hydrogel during injection. The large animal goat model of IVDD confirms that the granular hydrogel impedes disc height loss, increases proteoglycan production, and decreases collagen deposition [87].

Simple biomaterials or scaffolds may lack the ability to enhance cellular functions and direct stem cells to specific lineages. Therefore, biological factor-loaded biomaterials harness the power of active molecules to improve the efficacy of medical treatments. Pentosan polysulfate (PPS), a semi-synthetic GAG-like polysaccharide, has been proven to boost viability and induce MSCs differentiating to chondrocytes, and induce NP-like ECM production [88]. Frith et al. incorporated soluble PPS into polyethylene glycol and hyaluronic acid hydrogel system and encapsulated MPCs. The composites form NP-like tissue, particularly type Ⅱ collagen deposition. This hydrogel is immunologically tolerant using a rat subcutaneous implantation model [89]. Afterward, the research group determined that a covalently bound PPS possesses a strong potential to mediate IVD regeneration [90]. Pereira et al. attempted to add stromal cell-derived fator-1 (SDF-1) to an HA-poly (N-isopropylacrylamide) hydrogel to attract Hu-MSCs. The composite enhances the migration of MSCs into compromised discs in comparison to HAP or SDF-1 alone [91]. Tsaryk et al. combined TGF-β3 delivery by gelatin microspheres with collagen-low molecular weight hyaluronic acid hydrogel. Of note, the material promotes nasal chondrocytes and MSCs to expand and differentiate without triggering inflammation or inducing cytotoxicity [92]. Hyaluronic acid scaffold seeded with human Wharton's Jelly-derived MSC (WJ-MSC) expressing high levels of TGF-β receptor Ⅰ and TGF-β receptor Ⅱ significantly restore the disc hydration in a rabbit model compared to scaffold along or WJ-MSC-lowTR-seeded HA scaffold. The morphological and histological analyses are consistent with T2-weighted MRI analysis [93].

**Fig. 4:**
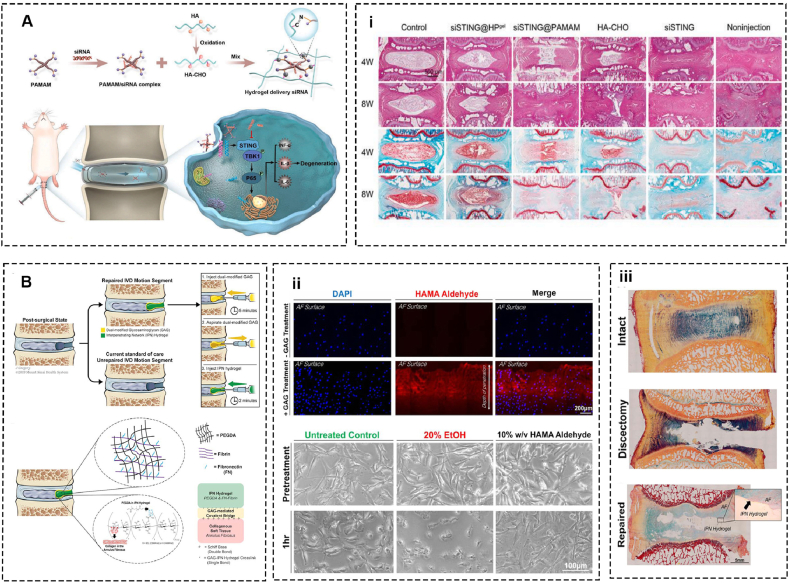
Hyaluronic acid-based biomaterials for intervertebral disc regeneration and repair. (A) Injectable self-healing hydrogel with continuous siRNA delivery property and (i) Representative H&E and Safranin O and Fast Green staining images demonstrate siSTING@HP^gel^ slowing the IVD degeneration progress (Adapted from Ref. [95]). (B) A two-part biomaterial adhesive strategy involving dual-modified glycosaminoglycan and interpenetrating network hydrogel for annulus fibrosus repair and (ii) Cross-sectional view of HAMA Aldehyde treated AF and phase-contrast images of HAMA Aldehyde treated AF cells. (iii) Picrosirius red/alcian blue staining (Adapted from Ref. [98]).

Targeting RNA molecules is a promising therapeutic strategy for IVDD. This approach focuses on modulating the expression and function of various RNAs including long noncoding RNAs (lncRNAs), circular RNAs (cirRNAs), and micro RNAs (miRNAs) to influence cellular processes including apoptosis, proliferation, and extracellular matrix remodeling in NPCs [94]. Recent studies demonstrate that stimulators of interferon genes (STING) are critical in diverse inflammatory and degenerative diseases. Chen et al. revealed that the STING signaling pathway is an intervention target for IVDD. A siSTING delivery hydrogel of aldehyde HA and poly (amidoamine)/siRNA complex is fabricated and the hydrogel system alleviates intervertebral inflammation and degeneration in the IVDD rat model [95] (Fig. 4A). Likewise, circSTC2 is elevated in degenerative NP tissue, and the silence of circSTC2 can restore the extracellular matrix anabolism and catabolism balance. Chang et al. grated circSTC2 silencing genes-loaded 1,2-dioleoyl-3-trimethylammonium-propane/cholesterol/1,2-dioleoyl-sn-glycero-3-phosphoethanolamine cationic liposomes on methacrylated hyaluronic acid microspheres. With the excellent merits including degradability, dispersibility, swellability, and injectability of HAMA microspheres, lipoplexes are persistently released over 27 days. This psh-circSTC2-lipo@MS enhances ECM anabolism and impedes catabolism in NPCs in vitro as well as in an in vivo rat IVDD model [96].

HA can be combined with different biomaterials to ameliorate pain and inflammation due to its anti-inflammatory properties. Fragmentation of ECM accompanies IVDD. However, research indicates that hyaluronic acid fragments induce inflammatory and catabolic mediators such as interleukin-1β, IL-6, cyclooxygenase-2, MMP-1, and MMP-13. Through siRNA intervention or antibody neutralization, it is suggested that hyaluronic acid fragments result in IVDD via the Toll-like receptor 2 signaling pathway [97].

#### 4.2.2 Applications of hyaluronic acid-based materials in AF regeneration

Currently, there are some challenges and limitations associated with using hydrogel systems for sealing annulus fibrosus defects. For example, many hydrogels exhibit poor adhesion to wet tissue and insufficient mechanical strength that may lead to potential dislodgement and failure to seal the defect effectively. Tyler et al. designed a combined regeneration system by integrating oxidized and methacrylated glycosaminoglycan and fibronectin-linked fibrin and poly (ethylene glycol) diacrylate. The former absorbs the latter interpenetrating network and binds this hydrogel to ECM in the IVD covalently. The results show that the dual-modified hydrogel presents excellent adhesive strength without any observable cytotoxic effects and demonstrates mechanical compliance that aligns with the herniation risk of the current clinical standard [98] (Fig. 4B). Interestingly, HA itself demonstrates an anti-inflammatory action in AF-defected and IFNα_2_β stimulated discs by downregulating IFIT3, ADATS4, and pro-apoptotic IGFBP3. Moreover, HA intervention increases collagen Ⅰ and aggrecan. Therefore, HA exhibits an anti-inflammatory property and matrix modulatory effect in annulus fibrosus [14].

#### 4.2.3 Applications of hyaluronic acid-based materials in combined therapy of NP and AF regeneration

Hyaluronic acid (HA)-poly (ethylene glycol) (PEG) composite hydrogels are a prominent area of research due to their unique properties. Jeong et al. used artificial neural network analysis to screen HA-PEG formulation to match NP and AF cells. It turns out that lower-molecular weight HA leads to the highest NPC and AFC numbers and higher sGAG production [99]. Based on the concept of IVD as integrity, Sloan et al. attempted to repair AF defect with riboflavin cross-linked high-density collagen gel and repair NP with modified HA hydrogel. Qualitative T2 MRI shows that combined therapies restore disc water content and preserve intact IVD morphology. Particularly, HA injection into NP recovers 35 % and 40 % of the effective instantaneous and equilibrium moduli [100]. Similarly, Mohd et al. confirmed that HA hydrogel attenuated IL-6 and IL-1β in degenerated IVD validated by upstream regulations of c-Fos, phosphorylated p38 MAPK, and phosphorylated NF-κB in both AF and NP and regulated ECM deposition, such as higher CS, collagen Ⅱ, and fibronectin, via Smad3-TGF-β1 signaling [101]. It has been implied that CS increases ECM production and correlates with peripheral sensory innervation and nociceptor. However, whether there exists direct action of chondroitin needs to be fully elucidated. Furthermore, HA hydrogel implantation inhibited injury-induced peripheral sensory hyperinnervation and pro-nociceptive TRPV1 and Trk-A expression in AF and NP tissues [101]. These results suggest that HA hydrogel has a potential therapeutic application and could be translated clinically in patients with discogenic back pain due to IVDD.

### 4.3 Cellulose

Cellulose is a linear polysaccharide in which D-glucose units are connected by β-(1,4) bonds [102]. Due to its superior mechanical properties and biocompatibility, nanoscale cellulose has garnered significant attention in recent years [103]. Cellulose nanomaterials can be classified into two types: cellulose nanostructure nanomaterials including cellulose microcrystals and microfibrils, and cellulose nano-objects including cellulose nanocrystals and cellulose nanofibrils [104]. In comparison to the former nanocelluloses, cellulose nanocrystals and cellulose nanofibrils exhibit elevated specific surface areas and a more uniform distribution of particle sizes, therefore promoting the formation of mechanically robust and spontaneously organized structures such as hydrogel [105].

#### 4.3.1 Applications of cellulose-based materials in NP regeneration

Cellulose-based scaffolds have remarkable merits such as non-immunogenicity, nontoxicity, strong biomechanical properties, as well as injectability and in situ gelling that, therefore, hold a potential for NP replacement [16]. Anna et al. first encapsulated NPCs in photocrosslinked carboxymethylcellulose (CMC) in which CMC was functionalized with methacrylate groups and the latter endow water solubility. Under normal pH conditions, the deprotonation of the carboxylic acid within the carboxymethyl groups leads to the negative charge of hydrogel that resembles the negatively charged glycosaminoglycans of ECM of NP. In addition, this polymer demonstrates support for NP cell viability with the occurrence of spherical cells and the composition of chondroitin sulfate proteoglycan [106].

Varma et al. developed a dual-polymer network hydrogel composed of methacrylated carboxymethylcellulose and methylcellulose (MC). The thermogelling MC facilitates the gelation of CMC in situ consistently within intervertebral discs. After injection into IVDs following discectomy, the CMC-MC composite is able to recover IVD height and biomechanical properties [107]. In a follow-up study to examine the sustaining stability of the structure, the CMC-MC hydrogel showed restoration of height and manifested similar herniation risk and fatigue resistance to those with standard nucleotomy. Additionally, the hydrogel injection only results in a limited foreign body reaction, evidenced by analyzing fibrous capsule formation and macrophage infiltration during a 12-week period [108].

Similarly, Gupta et al. introduced human MSCs encapsulated carboxymethylcellulose hydrogel stimulated with transforming growth factor-beta 3 (TGF-β3). This hydrogel system confirmed significant glycosaminoglycan and type Ⅱ collagen deposition that fit in with the ECM of NP [109]. Furthermore, the research group dug into the impact of the duration of TGF-β3 exposure on matrix metabolism and the best macromer concentration of CMC hydrogel. The results showed that a lower macromer concentration (1.5 % weight/volume) demonstrates stronger NP-like ECM elaboration and improved mechanical functionality and short-term TGF-β3 exposure augments sufficient ECM accumulation [110,111].

Prior investigations have mainly focused on small animal models and short-term outcomes spanning just for a few weeks. Extensive long-term studies in relevant large animal models are imperative for translating into clinical application. An injectable chitosan CMC hydrogel system supplemented with adipose-derived stem cells (ASCs) was evaluated in an ovine model for 12 months. Of note, this scaffold proved a long-term stabilization that alleviated IVDD and sustained intervertebral height in comparison to injured and untreated IVDs [112]. Incorporation of cellulose nano-objects with synthetic polymer (e.g. poly (ethylene glycol) (PEG)) matrix can produce hydrogels that have improved mechanical stability. Schmocker et al. developed a photopolymerized PEG dimethacrylate (MA) cellulose nanofibrils (CNFs) composite hydrogel. The material was finely tailored to fit the elastic modulus and water content of native NP. By utilizing a newly designed device to realize in situ injection and photopolymerization, the composite hydrogel is capable of rebuilding disc height within an ex vivo bovine organ model even after 0.5 million loading cycles and histological analyses indicate a superb tissue integration of this hydrogel implant [113].

#### 4.3.2 Applications of cellulose-based materials in AF regeneration, combined therapy of AF and NP

Celluloses are usually used as reinforcement by incorporating cellulose nanofibrils and nanocrystals into synthetic or natural polymers. Pereira et al. fabricated an injectable nanocellulose reinforced methacrylated gellan-gum (GGMA) composite hydrogel for AF substituting. The hydrogel system manifests compressive modulus consistent with native AF tissue and improved matrix entanglement with augmented scaffold stiffness. In vitro experiments indicated that nanocomposite hydrogel enhanced cell survival of encapsulated bovine AFCs and maintained AFCs physiological morphology [114].

**Fig. 5:**
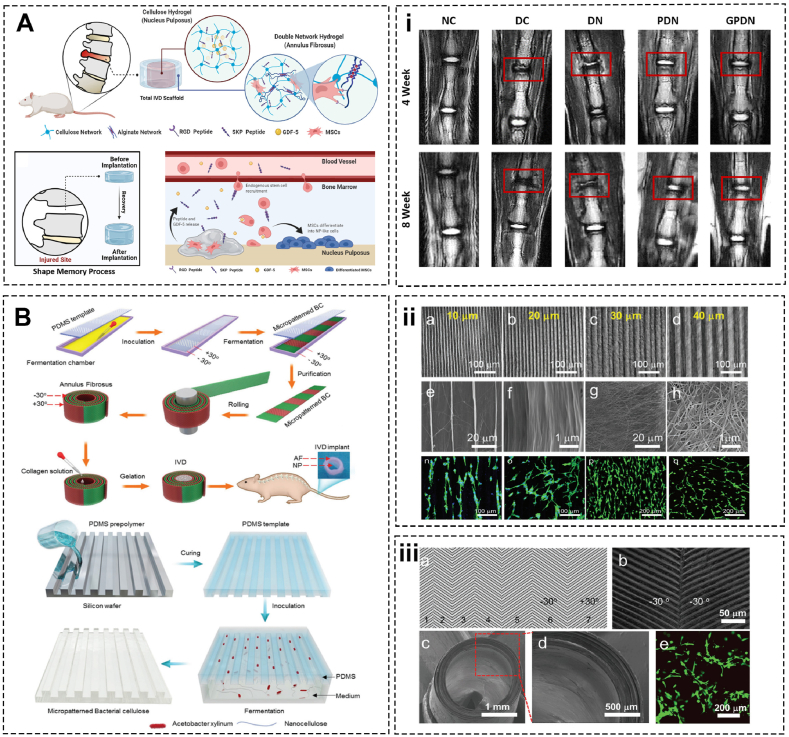
Cellulose-based biomaterials for intervertebral disc regeneration and repair. (A) Peptide-functionalized (RGD and SKP peptides) cellulose/alginate double network hydrogel and (i) T2‐weighted MRI imaging of the caudal spine (marked with red boxes) (Adapted from Ref. [115]). (B) Reverse reconstruction and bioprinting of bacterial cellulose-based functional IVD consist of micropatterned bacterial cellulose aligned in ± 30° direction as AF and gelated type Ⅱ collagen as NP and (ii) a-d) Phase contrast microscopy imaging of microgrooves. e-h) SEM image for the BC membrane. n-q) Alexa Fluor 488 phalloidin/Hoechst staining. iii) a,b) Designed pattern of the AF template in opposing orientations ( ± 30°) within adjacent lamellae. c,d) SEM transection images for AF. e) Live/dead staining of APCs (Adapted from Ref. [116]).

Individual NP and AF regeneration strategies have been broadly developed to alleviate IVDD. However, the integrity of IVD implies that individual therapy is insufficient and emphasizes the importance of combined AF and NP therapies. Hydrogels based on a cellulose-alginate dual network for the AF and a cellulose hydrogel for the NP are designed to replace IVD. This scaffold increased endogenous MSC homing and adhesion due to functionalized SKP peptide and RGD (Arg-Gly-Asp) peptide and induced MSCs differentiation to chondrocyte and NP-like phenotype with the delivery of growth differentiation factor 5 (GDF-5). Moreover, many other advantageous merits include enhanced compressibility, shape retention capability and comparable biomechanical strength. Of particular, the rat caudal IVDD model demonstrated that the implant impeded the degeneration progress and significantly reestablished both AF and NP [115] (Fig. 5A). Similarly, Yang et al. created a more complex IVD implant that consists of type Ⅱ collagen-based NP and bioprinted bacterial cellulose-based AF. The former involves rat NPCs while the multilamellar bacterial cellulose layers with micropattern in ± 30° alternative direction contain AFCs. Animal studies confirmed that this tissue-engineered IVD can integrate with the adjacent vertebral and withstand tensile stresses for as long as 3 months [116] (Fig. 5B).

### 4.4 Alginate

Alginate, a naturally occurring polysaccharide extracted from brown seaweed and certain bacteria, features linear chains of repeating units of β-D-mannuronic acid and α-L-guluronic acid, which are connected together by 1,4-glycosidic bonds [117]. Previous studies have shown that Alginate can reduce ROS levels in cells by chelating metal ions. Moreover, phosphorylation of IκB-p65 complex is downregulated by inhibiting nuclear translocation of P65. It may also reduce phosphorylation of P-Erk1/2 and P-JNK in a concentration-dependent manner [118]. Its unique characteristics, determined by the ratio of mannuronic acid to guluronic acid, allow it to form hydrogels that can be tailored for specific uses, including cell encapsulation, delivering drugs, wound repair, and scaffold fabrication [119]. The ability to customize its mechanical and biological properties makes alginate an essential material for advancing regenerative medicine and other therapeutic applications.

#### 4.4.1 Applications of alginate-based materials in NP regeneration

Due to its excellent biocompatible property, alginate hydrogel is one of the most extensively employed biomaterials and has been shown to maintain a juvenile rounded morphology [120]. Different gelling crosslinker concentrations may lead to distinct mechanical strength and degradability. Kalaf et al. determined that a ratio of 60 mM calcium carbonate to 120 mM glucono-δ-lactone is identified as having the most optimal properties compared to 2 % alginate crosslinked with calcium chloride. Injecting in situ gelling 2 % alginate into an enzyme-induced comprised IVD reestablishes functionality by reducing height reduction during prolonged cyclic loading and remains contained within the disc with no leakage at the injection area [121].

Simple alginate hydrogel may present insufficient to rebuild compromised IVD. BMSCs or bone marrow aspirate concentrate (BMAC) loaded ultra-purified alginate (UPAL) gels have been delineated as effective in the repair of IVD defects [122]. Similarly, Tilotta et al. trapped human NPCs in alginate spheres and then stimulated them with Wharton's Jelly MSC-derived extracellular vesicles. The intervention leads to cell content increase and cell death decrease. Nitrites, increasing when NPCs face oxidative stress, drop drastically after extracellular vesicle treatment. Moreover, proteoglycan content, indicated by Alcian blue staining, is upregulated. Therefore, extracellular vesicles ameliorate IVD degeneration and inflammation while enhancing ECM production [123]. Traditional simple alginate hydrogels lack mechanical strength when implanted into animal models. Zeng et al. seeded MSCs in alginate and reinforced with macroporous poly (ethylene glycol) diacrylate (PEGDA) microcryogels (PM). The system creates 3D microscale niches and shows improved elasticity. Reinforced alginate hydrogel impedes cell leakage and enhances cell survival. In a canine animal model, PM-alginate hydrogel rescues IVDD after 6 months compared to other treatments [124].

**Fig. 6:**
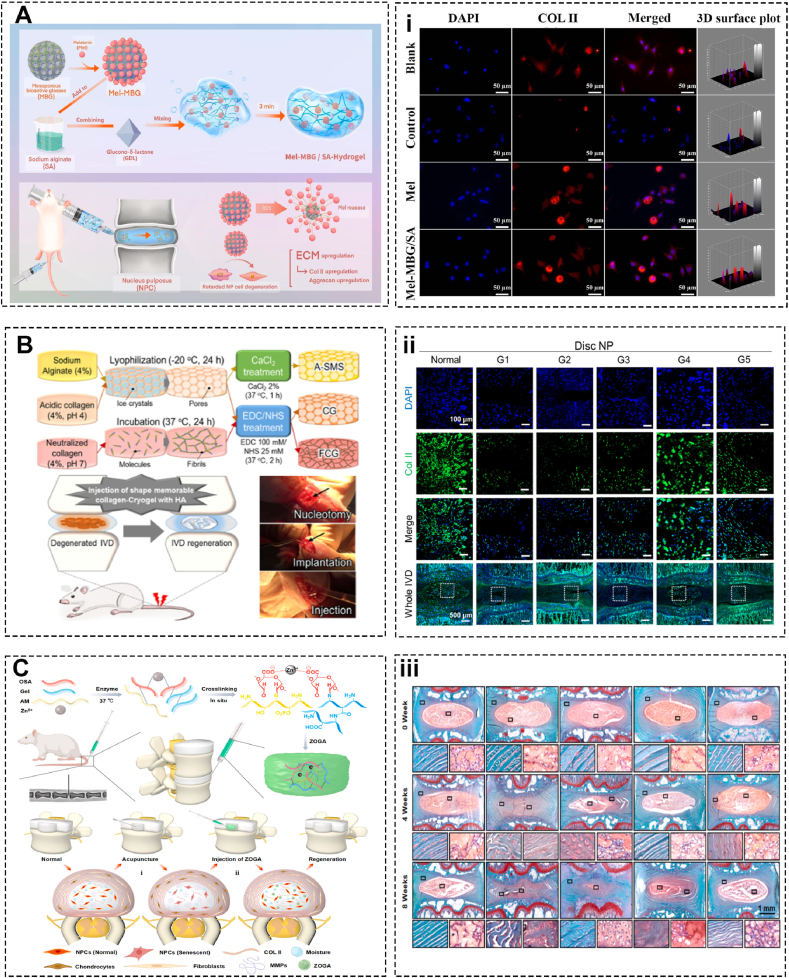
Alginate-based biomaterials for intervertebral disc regeneration and repair. (A) Injectable mesoporous bioactive glass/sodium alginate hydrogel loaded with inflammation suppressor melatonin and (i) Immunofluorescence analysis of COL-II expression in NPCs (Adapted from Ref. [128]). (B) Shape-memory collagen/alginate scaffold and in vivo procedures including nucleotomy, implantation of SMSs, and injection of HA, and (ii) Immunofluorescent analyses of collagen type II (Adapted from Ref. [129]). (C) Engineered high-strength biohydrogel consists of oxidized sodium alginate, gelatin, antagomir-204–3p, and Zn^2+^ as a multifunctional platform to deliver nucleic acid. iii) SF staining images of IVD (Adapted from Ref. [130]).

Preserving the juvenile phenotype of NPCs is crucial for IVD regeneration. Syndecan binding peptide (AG73) has been shown to promote NPCs adhesion and the RGD peptide motif has been shown to modulate the mechanotransduction in NPCs [125,126]. Tan et al. designed alginate hydrogels combined with AG73 and cyclic RGD. The results show that peptides hydrogel demonstrate high cell viability, ECM synthetic property, and induce NPC phenotype, like N-Cadherin [26]. With the aim of regulating local inflammatory microenvironment, Jiang et al. developed a hydrogel composite composed of sodium alginate, silicate ceramics, and poly (N-isopropylacrylamide). Poly (N-isopropylacrylamide) crosslinks in response to body temperature and forms an interpenetrating network with sodium alginate. Silicate ceramics (SC) release calcium ion that promotes alginate sodium crosslink. Magnesium ion from SC contributes to M2 macrophage polarization and regulates the immune microenvironment in a controlled manner, hence enhancing cell matrix production in which collagen Ⅱ and aggrecan are elevated [127]. Similarly, Wu et al. add melatonin, recently shown to possess antioxidant and anti-inflammatory action, to mesoporous bioactive glasses (MBG) and sodium alginate (SA) composite hydrogel. MBG provides sufficient mechanical strength and acts as a melatonin reservoir to facilitate continuous release. The melatonin-MBG-SA hydrogel ameliorates IVD inflammation including TNF, ADAMTS5, MMP-3, and MMP-13 triggered by IL-1β by interacting with cell membrane melatonin receptors (MT1 and MT2). Additionally, melatonin enhances cell viability and facilitates continuous cell differentiation of MSCs by scavenging ROS and activating Nrf2 signaling. In vivo rat model shows that this composite hydrogel effectively inhibits inflammation and restores mechanical stability [128] (Fig. 6A). Koo et al. used an alginate construct loaded with collagen and hyaluronic acid to form a shape-memory structure (SMS). The findings indicate that the SMS is effective in reducing pain, maintaining hydration, and preserving the structural integrity and function of the IVD matrix, together with supporting cell viability and the production of essential matrix components [129] (Fig. 6B).

Natural sodium alginate is usually gelated by calcium chloride in which endotoxins are relatively high with regard to in vivo implant. Ultra-purified alginate (UPAL) has been suggested to have a favorable lower concentration of endotoxins. Of note, UPAL hydrogel shows superior biomechanical characteristics and does not exhibit material extrusion following discectomy. Furthermore, UPAL gel enhances anabolism in NP, and maintains the moisture of IVDs in rabbits and sheep models. Surprisingly, the gel recruits endogenous NP progenitor cells in NP tissues [131]. Subsequently, the same research group encapsulated BMSCs into UPAL gel. Co-culture of NPCs and BMSCs demonstrates elevated ECM synthesis, phenotypic markers, and bioactive factors. Combined BMSCs and UPAL hydrogel display augmented potential to rebuild degenerated IVDs [132]. With the aim to administrate into the IVD by quick injection in situations involving persistent low back pain, a mixture of rapidly expanding clones of human MSCs and non-gelling UPAL solution is shown to impede inflammatory cytokine production and improve nociceptive behavior of rats [133]. Of note, these UPAL gels are presently carried out in an initial human clinical trial in young patients, aged 20 to 40, after discectomy [134].

Utilizing bioactive materials to deliver nucleic acids proposes a promising treatment for IVD regeneration. Antagomir-204–3p (AM) is capable of restraining NPCs apoptosis with lower immunogenicity. Chen et al. used zinc-oxidized sodium alginate-gelatin (ZOG) to distribute AM in which the former is able to crosslink with NP. In vivo results demonstrate that ZOG-AM rejuvenates the intervertebral disc height, sustains disc hydration, and upholds tissue integrity [130] (Fig. 6C).

#### 4.4.2 Applications of alginate-based materials in AF regeneration

Cell therapy holds potential for IVD repair, but the biomaterials used for cell delivery, which can seal annulus fibrosus defects and restore biomechanical function, often exhibit subpar biological performance. With the aim to keep the mechanical and biological performance balance, Panebianco et al. fabricated a composite hydrogel system utilizing AFCs seeded-oxidized alginate microbeads (MBs) loaded into fibrin (FibGen) hydrogels through genipin-crosslinking. AFCs encapsulated in microbeads demonstrate high cell survival, which is vital for long-term healing while high-modulus fibrin hydrogels provide immediate stabilization to degenerated IVDs. Subsequently, the FibGen-MB hydrogels are functionalized with RGD peptides and demonstrate significantly reduced AFC apoptosis and maintained phenotypic gene expression. The cell-encapsulated FibGen-MB-RGD hydrogels are assessed during a prolonged bovine caudal IVD organ model and exhibit a low risk of herniation [135].

#### 4.4.3 Applications of alginate-based materials in combined therapy of AF and NP

Inflammatory cytokines including interleukin-1 and tumor necrosis factor α are elevated in IVDD and participate in inflammation cascade and ECM degradation. Kakutani et al. determined that concurrent treatment inhibits IL-1β and TNF-α production of NPCs, AFCs, and explants encapsulated in alginate by using IL-1 receptor antagonist (IL-1Ra) and soluble tumor necrosis factor receptor-1 (sTNFR1) and promotes proteoglycan and collagen production in the NPCs and AFCs. Additionally, NPCs and AFCs produce these two cytokines in autocrine or paracrine manners via feedback regulation [136]. Du et al. designed a biomimetic annulus fibrosus and nucleus pulposus composite consisting of circumferential poly (ε-caprolactone) microfibers laden with AFCs and an NPCs encapsulated alginate hydrogel core. The artificial IVD exhibits increased ECM deposition and organization, as well as strengthened mechanical strength [137].

### 4.5 Chitosan

Chitosan is a biopolymer obtained from chitin, an abundant polysaccharide present in the exoskeletons of crustaceans and in fungal cell walls. Chitosan is formed by deacetylating chitin, a process that removes acetyl groups from the polymer chain [138]. Structurally, chitosan is composed primarily of β-(1–4)-linked D-glucosamine units (deacetylated units) and N-acetyl-D-glucosamine units (acetylated units). The extent of deacetylation (DD) determines the ratio of glucosamine to N-acetylglucosamine units in the polymer chain [139]. An acid hydrolysis product of chitosan, chitosan oligosaccharide (COS), has been shown to have significant anti-inflammatory properties. It can inhibit LPS-induced inflammatory cytokines such as TNF-α and IL-6 and nitric oxide secretion in a dose-dependent manner, thereby suppressing phosphorylation of JNK and translocation of p65, a subunit of NF-κB, into the nucleus [140]. The unique combination of biocompatibility, biodegradability, antimicrobial properties, and ability to form films and gels of chitosan makes it a versatile biomaterial in diverse fields, for example, biomedicine, food technology, agriculture, and industrial applications [141].

#### 4.5.1 Applications of chitosan-based materials in NP regeneration

Based on the specific hypothesis that the cationic chitosan, due to its positive charge, creates an ideal environment for the entrapment of large quantities of newly synthesized anionic proteoglycans. Roughley et al. found that various formulations of cell-encapsulated chitosan hydrogels preserved a considerable portion of the proteoglycan generated by NPCs inside the hydrogel matrix, therefore suggesting the capability of chitosan as a platform for cell-loaded supplementation aimed at restoring disc function and structure [142]. Furthermore, thermosensitive chitosan-glycerophosphate hydrogel has been proved to promote MSCs secreting proteoglycans and collagens in a proportion that is similar to that of nucleus pulposus cells [143]. Among these different formulations, a physical crosslinked hydrogel that contains 2 % (w/v) chitosan, 0.075M sodium hydrogen carbonate, and 0.1M β-glycerophosphate proves to simulate the human nucleus pulposus tissue in mechanical properties and stimulates the highest production and retention of glycosaminoglycans by the loaded cells within the gel [144]. Through N-hexanoylation of glycol chitosan, Li et al. developed a new hydrogel system with tunable thermos-sensitivity and enhanced stability. Biochemical serum profiles and histological examination of hexanoyl glycol chitosan-treated rats demonstrate excellent biocompatibility compared to saline-treated rats. The hydrogel maintains its stability for over a month in an ex vivo porcine system, supporting its potential use in IVDD [145].

**Fig. 7:**
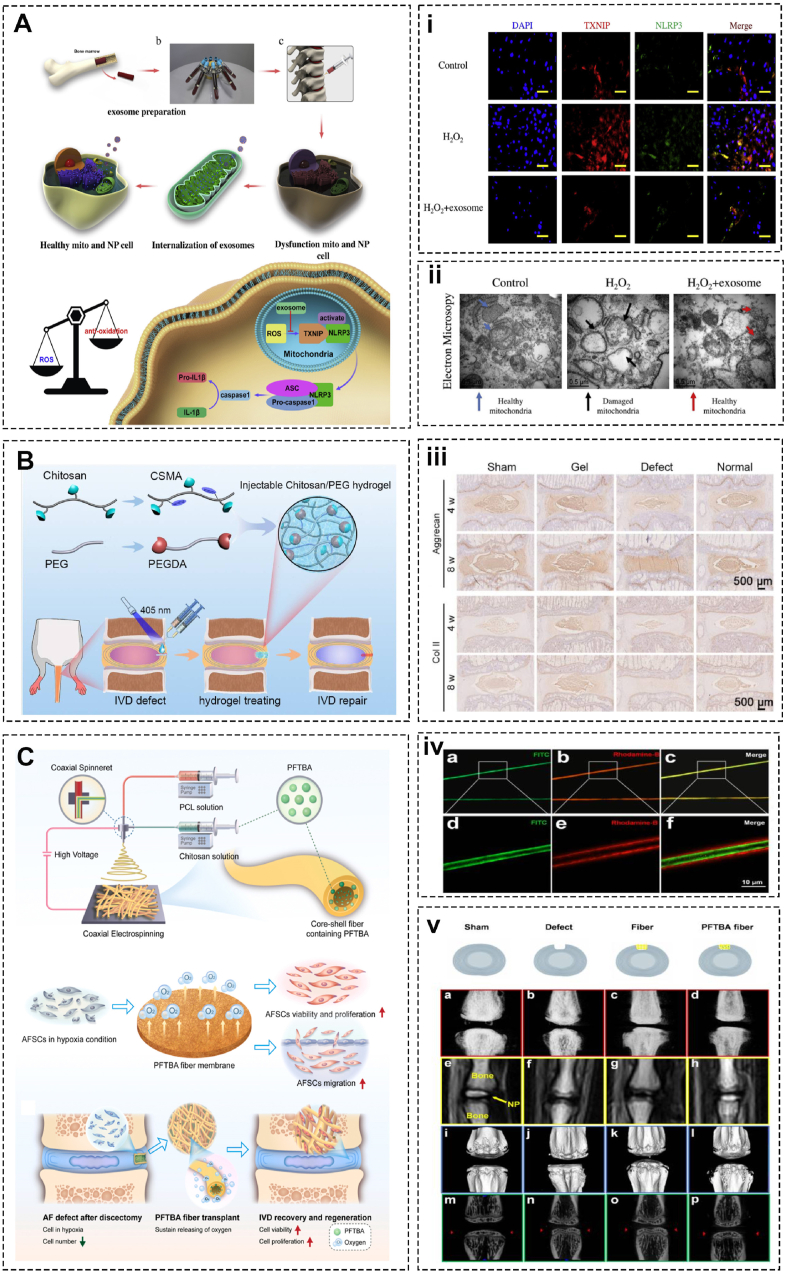
Chitosan-based biomaterials for intervertebral disc regeneration and repair. (A) Anti-oxidant and anti-inflammatory effect of mesenchymal stem cell-derived exosomes derived from C57BL/6 mice in an IVDD rabbit model and possible signal pathway. (i) Immunocytochemistry of NLRP3 and TXNIP. (ii) TEM to evaluate the microscopic structure of mitochondria (Adapted from Ref. [146]). (B) In situ forming an injectable Chitosan/PEG hydrogel shaped by Schiff base reaction with light exposure under 405 nm LED and (iii) Immunohistochemistry staining of Aggrecan and collagen II (Adapted from Ref. [150]). (C) Core-shell oxygen-releasing fibers via coaxial electrospinning, actions of PFTBA on the viability and functions of AFSCs, and implantation of the PFTBA core-shell scaffold in AF repair after discectomy and (iv) Fluorescence microscopy of the core-shell structure. (v) X-ray, MRI and Micro-CT (Adapted from Ref. [154]).

Inflammation is critical in the pathogenesis of IVDD, with macrophages and inflammatory pathways significantly contributing to disc degeneration [6]. Chronic low-grade inflammation and cellular senescence further exacerbate the condition. Targeting inflammatory processes offers a promising strategy for the treatment and management of IVDD [146] (Fig. 7A). Diclofenac, one of non-steroidal anti-inflammatory drugs (NSAIDs), has been reported to impede the pro-inflammatory polarization of human macrophages. Teixeira et al. evaluated the anti-inflammatory action of nanoparticles (NP) of chitosan and poly-(γ-glutamic acid) with diclofenac (Df). After internalization of IVD cells, nanoparticles inhibit IL-6, IL-8, PGE2, and MMPs expression and upregulate aggrecan and type Ⅱ collagen production [147]. Subsequently, the same research group improved the nanocomplexes at pH 7.4 instead of 5.0 in that they found NPs under such an environment promote ECM anabolism including type Ⅱ collagen and sulfated glycosaminoglycan production [148]. Furthermore, via systematic analysis of immune cell responses, the results show that pro-inflammatory intradiscal administration leads to aggravated IVD lesions, as indicated by increased CD4^+^ T cells (CD4^+^CD8-TcR + CD161-) in the lymph nodes and blood and more pro-inflammatory cytokines, such as IL-1β, IL-6, and COX-2. In contrast, intradiscal anti-inflammatory administration slows the breakdown of the injured IVD and is associated with elevated systemic myeloid cell level (CD11b + MHCⅡ+) [149].

Traditionally, simple chitosan hydrogel possesses low mechanical strength, therefore limiting its use to biomedical applications. Huang et al. introduced a fast in situ crosslinking injectable chitosan/polyethylene glycol (PEG) hydrogel with improved machinal strength via incorporating a photo-crosslink of methacrylate and Schiff base reaction between these two components simultaneously. In vitro experiments have proved the biocompatibility and proliferation of NPCs. Micro-CT and MRI at 4 and 8 weeks claim promising effects on retarding the IVDD in rat tails [150] (Fig. 7B). Similarly, Adoungotchodo et al. fabricated a chitosan-based hydrogel incorporating gelatine and Link N (LN), a peptide that naturally occurs in cartilage and the ECM of IVD. Rheological and compressive mechanical tests demonstrate that gelatine mildly affects hydrogel gelling but significantly strengthens mechanical properties in compression. Moreover, LN induces notably high glycosaminoglycan production in degenerative environments [151]. Li et al. used poly (l-lactic acid) to enhance mechanical strength by distributing the former throughout chitosan scaffolds with human umbilical cord mesenchymal stem cells encapsulated. In vitro cell culture claimed that this hydrogel network is suitable for cell adhesion and proliferation. After being inserted for lumbar fusion, chitosan/PLLA enchance bone connectivity between adjacent vertebrae and, in the meantime, aids in the regeneration of annulus fibrosus tissue [152].

Based on the distinctive biomechanical characteristics of native cellulose nanofibers (CNFs), a 3D printed, nature-inspired CNF-filled CHI hydrogel was proposed in which the former brings in essential mechanical reinforcement, whereas the latter acts as a biocompatible matrix that promotes cell growth. Because chitosan and cellulose are purely natural and are fabricated without any modification of components and the addition of a chemical crosslinker, this integrated hydrogel matrix supports 3D cell seeding with superior cell viability. The composite hydrogel opens up new possibilities for designing demanding engineered tissue of intervertebral discs [153].

#### 4.5.2 Applications of chitosan-based materials in AF regeneration

To date, there has been limited information available regarding strategies to address the challenge of hypoxia in annulus fibrosus (AF) defect sites. However, in a recent study, researchers have explored the use of perfluorotributylamine (PFTBA) core-shell fibers (10 % chitosan, chitosan: PCL, 1:6) fabricated via coaxial electrospinning. This approach aims to fabricate an oxygen-releasing platform designed to promote natural reconstruction processes in the annulus fibrosus following resecting disc. In vitro studies demonstrate that hydrogels facilitate proliferation, migration and ECM synthesis in annulus fibrosus stem cells under hypoxic conditions and in vivo studies show notable improvements in vertebral spacing and structural unity observed in animal models treated with these oxygen-releasing biomaterials [154] (Fig. 7C).

#### 4.5.3 Applications of chitosan-based materials in combined therapy of AF and NP

Tissue-engineered intervertebral discs (TE-IVDs) have been considered an effective therapeutic solution for treating IVDD. Yang et al. developed an artificial IVD employing chitosan hydrogel to mimic the central nucleus pulposus region, enclosed by a poly (butylene succinate-co-terephthalate) fiber layer to represent the inner annulus fibrosus and employing a poly (ether ether ketone) ring to simulate the outer annulus fibrosus with IVD cells seeded. The novel tissue-engineered IVD establishes a conducive setting for supporting the development of IVD cells. Overall morphology and biological functions of the scaffolds closely resemble those of natural porcine IVDs [155]. Afterward, Yuan et al. constructed similar TEIVDs using poly (butylene succinate-co-terephthalate) copolyester as inner annulus fibrosus (IAF) while solid PBST as outer annulus fibrosus (OAF), and using chitosan as NP. AF and NP cells are loaded onto the corresponding scaffolds. Of note, the IVD cells express cell-specific extracellular matrix, therefore suggesting that TE-IVD is biologically functional. X-ray and MRI examinations indicated that the intervertebral disc space was maintained 4 weeks later after TE-IVD was engrafted into New Zealand white rabbits [156].

## 5 Conclusions and perspectives

Polysaccharide-based materials hold significant promise as therapeutic agents for IVDD, offering a versatile and biocompatible platform for tissue regeneration. Of these materials, hyaluronic acid and chondroitin sulfate, two key components of the NP's extracellular matrix, possess the strongest ability to stimulate the nucleus pulposus environment with the ability to retain water and resist compressive forces. Chitosan has superior adhesivity to alginate, which has moderate adhesivity, and hyaluronic acid. With regard to biomechanical strength, alginate stands out with its tunable mechanical strength and stability. Moreover, compared to other inorganic materials or synthetic polymers which may require harsher processing conditions and yield harmful byproducts, polysaccharides can be processed under mild conditions such as low temperature, neutral pH and degrade via enzymatic hydrolysis. In the meanwhile, polysaccharides possess excellent bioactivity and inherent hydrophilic properties to retain hydration similar to native NP tissue. Of note, while proteins like collagen and synthetic polymers like PLGA offer tunability, polysaccharides like HA or chitosan can reach a unique balance of bioactivity and mechanical properties. To conclude, these polysaccharide-based biomaterials, including hyaluronic acid, chitosan, and alginate have demonstrated the ability to mimic the extracellular matrix of the NP, support cell survival, and enhance matrix synthesis, making them ideal candidates for disc repair and regeneration. Their capacity to deliver bioactive molecules directly to the degenerative site as well as their cost-effectiveness further amplifies their therapeutic potential.

Nevertheless, several challenges still remain despite of all these advances. The intricate biomechanics of the IVD necessitate the use of materials that are capable of providing support for cellular functions while simultaneously withstanding the mechanical loads and stresses that are inherent to the spinal environment. The AF and NP are anatomically and functionally interdependent and the integrity of NP and AF in IVD are needed to be considered. For instance, a weakened AF may lead to NP herniation, while a degenerated NP can lead to AF collapse. Herein, the combined therapy targeting both the NP and AF is critical for achieving comprehensive and durable outcomes. Furthermore, the transition of these materials from laboratory to clinical settings requires comprehensive investigations into their long-term biocompatibility, degradation profiles, and potential immunogenic responses. The development of injectable and minimally invasive polysaccharide-based systems is also of great importance in order to ensure clinical applicability and patient compliance.

In the future, the integration of advanced technologies, including three-dimensional bioprinting, nanotechnology, and gene editing, may provide new avenues for enhancing the efficacy of polysaccharide-based therapies. Polysaccharide-based materials can be customized to suit the specific requirements of both AF and NP, for instance, customized stiffness and elasticity through chemical modifications, functionalized polymer grafts, or in combination with other materials to fabricate composites, which may pave the way for meeting combined AF and NP therapy. 3D bioprinting can facilitate the precise fabrication of complex disc structures, while nanotechnology may be employed to design nanoparticles for targeted drug delivery within the disc environment. Gene editing technologies, such as CRISPR, may facilitate the modification of cells encapsulated within these hydrogels to produce specific proteins or growth factors that enhance tissue regeneration.

In conclusion, polysaccharide-based materials represent a promising and adaptable approach for the therapy of IVDD. Continued research and technological integration are essential to overcome current limitations and realize their full potential in clinical settings. Advancing these materials and strategies may facilitate the development of effective, long-lasting solutions for patients suffering from intervertebral disc degeneration.

## CRediT authorship contribution statement

**Xin Wang:** Writing – original draft, Software, Resources. **Yixue Huang:** Writing – original draft, Software, Resources. **Yilin Yang:** Resources. **Xin Tian:** Resources. **Yesheng Jin:** Resources. **Weimin Jiang:** Funding acquisition, Conceptualization. **Hanliang He:** Writing – review & editing, Funding acquisition, Conceptualization. **Yong Xu:** Writing – review & editing, Funding acquisition, Conceptualization. **Yijie Liu:** Writing – review & editing, Funding acquisition, Conceptualization.

## Declaration of competing interest

The authors declare that they have no known competing financial interests or personal relationships that could have appeared to influence the work reported in this paper.

## Data Availability

No data was used for the research described in the article.
